# A Prospective, Observational, Open-Label, Non-comparative, Multi-center Study for the Evaluation of Efficacy and Safety of Levonadifloxacin (Intravenous and Oral) in Patients With Community-Acquired Bacterial Pneumonia

**DOI:** 10.7759/cureus.76315

**Published:** 2024-12-24

**Authors:** Abhijit Telkhade, Chintan Patel, Rahul Kumar Rathode, Bhanu Singh, Shrikant Deshpande, Balaji More, Shubhangi Deshpande

**Affiliations:** 1 Respiratory Medicine, Shree Ashirwad Hospital, Mumbai, IND; 2 Respiratory Medicine, Hansa Clinic and Hospital, Daman and Diu, IND; 3 Respiratory Medicine, Charak Hospital and Research Centre, Lucknow, IND; 4 Respiratory Medicine, Midland Healthcare and Research Centre, Lucknow, IND; 5 Respiratory Medicine, Ashirwad Hospital, Thane, IND; 6 Respiratory Medicine, Mahatma Gandhi Medical College and Research Institute, Puducherry, IND; 7 Respiratory Medicine, Gujarat Medical Education and Research Society of Department of Health and Family Welfare (GMERS) Medical College and General Hospital, Vadodara, IND

**Keywords:** cabp, clinical success, levonadifloxacin, microbial cure, pneumonia

## Abstract

Background

Community-acquired bacterial pneumonia (CABP) is associated with a substantial healthcare burden. The emergence of multi-drug resistance in *Streptococcus pneumoniae, Haemophilus influenzae, and Moraxella catarrhalis* is becoming an increasing concern in the management of CABP. This study aims to evaluate the efficacy and safety of levonadifloxacin in the treatment of CABP, focusing on both oral and intravenous (IV) therapy.

Materials and methods

This prospective, post-marketing, multi-center, observational, investigator-initiated real-world study was conducted in adult CABP patients of any sex at eight healthcare facilities (Shree Ashirwad Hospital, Mumbai, Hansa Clinic & Hospital, Daman and Diu, Charak Hospital and Research Centre, Lucknow, Midland Healthcare & Research Centre, Lucknow, Ashirwadh Hospital, Thane, Mahatma Gandhi Medical College and Research Institute, Puducherry, GMERS Medical College and General Hospital, Vadodara, and KD Hospital, Ahmedabad) across India after obtaining written informed consent from the subjects. Basic demographic data including the treatment details were collected. The efficacy was assessed in terms of the clinical outcome on the fourth day after the first dose, at the end of treatment/EOT (seventh day to 14^th^ day after the first dose), and at test of cure/TOC (7±1 days after EOT) as well as microbiological outcome at the EOT. Evaluation of safety was based on adverse events (AEs) reported, vital signs and systemic examination findings, laboratory evaluation, and electrocardiograms (ECGs) collected during the study.

Results

Of the 101 patients, nine patients did not receive the drug and were excluded from the study. The intent-to-treat (ITT) analysis set consisted of 92 patients out of which 74 patients were included in the modified ITT (mITT) dataset as follow-up data were not available for 18 patients. Of the 74 patients, 51 patients (68.9%) were male, 22 patients (29.7%) were female and one patient (1.4%) was transgender. Of all the patients, 49 (66.2%) patients received oral therapy, eight (10.8%) received IV therapy and 17 (23.0%) received IV therapy followed by oral levonadifloxacin therapy. On the fourth day after the first dose, clinical improvement was reported in 49/74 (66.21%) subjects, and clinical cure was reported in 7/74 (9.45%) patients. At the end of treatment/EOT, clinical cure was reported in 69/71 (97.18%) subjects, and microbiological cure was reported in 67/71 (94.36%) subjects. At test of cure/TOC, clinical cure was reported in 63/63 (100%) subjects. No significant changes in vital parameters, laboratory parameters, or 12-lead ECG were observed with levonadifloxacin therapy during the study. Seven subjects [n=7/92 (7.60%)] were reported to have experienced the AEs during the study period. AEs included nausea, vomiting, myalgia, headache, and rash. All events were mild in severity and resolved without sequelae.

Conclusions

Levonadifloxacin administered by oral and/or IV route showed promising efficacy and safety outcomes in hospitalized patients or patients receiving outpatient therapy for CABP.

## Introduction

Lower respiratory tract infection, especially community-acquired pneumonia (CAP), is the second leading cause of hospitalization and death in all age groups, especially children under five years and the elderly [1,2]. Respiratory infections including CAP are common in India and contribute about 23% of the global pneumonia burden and 36 % of the World Health Organization regional burden [3]. CAP is majorly caused by bacteria (community-acquired bacterial pneumonia; CABP) and viruses and seldom by fungi. Various bacterial pathogens that cause CABP are *Streptococcus pneumoniae, Haemophilus influenzae, Moraxella catarrhalis, *and* Staphylococcus aureus,* and the more difficult-to-culture atypical pathogens (*Legionella pneumophila, Mycoplasma pneumoniae *and* Chlamydophila pneumoniae*). Among them, *S. pneumoniae* is the most commonly identified bacterial pathogen [1,4].

The main treatment objectives in the CABP include microbiological purge, clinical improvement, minimizing the hospital stay, and preventing relapse [5]. In the US, despite the availability of cutting-edge diagnostics, microbiology etiology of CABP is established only in 30 to 40% of CABP cases. This proportion could be much lower in India. As a result, antibiotic treatment for CABP is initiated on an empirical basis. Regardless of the types of clinical presentations, prompt initiation of antibiotic therapy is needed in most cases of CABP [6].

The Infectious Diseases Society of America and the American Thoracic Society (IDSA/ATS) [7], the British Thoracic Society (BTS) [8], and the Indian Chest Society and the National College of Chest Physicians (ICS/NCCP) [9] provide the antibiotic treatment recommendations for CABP. About 80% of CABP cases are treated in outpatient settings and the rest are hospitalized to administer intravenous (IV) antibiotics. For CABP patients without comorbidities and risk factors, the commonly followed approach is to deploy an oral monotherapy of a β-lactam (amoxicillin or oral cephalosporin) or a macrolide (azithromycin or clarithromycin) or a tetracycline (doxycycline). For those with comorbidities and risk factors and also in regions with high macrolide-resistance rates, combination therapy is the norm. Such combination therapy is constituted of a β-lactam (amoxicillin/clavulanic acid or oral cephalosporin) plus macrolide or doxycycline. This combination therapy is warranted to cover macrolide-resistant *S. pneumoniae* as well as atypicals. Alternatively, monotherapy with older fluoroquinolone (levofloxacin, moxifloxacin, or gemifloxacin) is considered but largely not preferred in India. This is due to the risk of resistance selection in Mycobacterium tuberculosis as many CABP patients might be also suffering from underlying tuberculosis. Moreover, fluoroquinolone treatment may result in a false negative result in diagnosing tuberculosis. Thus, there is an unmet need for a safe antibiotic that is comprehensively active against all CABP pathogens and possesses pharmacokinetic/pharmacodynamic features commensurate with CABP treatment. The antibiotic treatment for hospitalized CABP patients also requires a combination approach. In recent years even though there are existing antibiotics and chemotherapeutic agents, multi-drug resistance problems have increased globally among various microorganisms due to the overuse and/or abuse of antibiotic drugs and the lack of new drug discovery programs being undertaken by pharmaceutical companies [10,11]. Cephalosporins are reported to lack activity against atypical respiratory pathogens, which requires concomitant administration of another suitable antibiotic active against atypical pathogens. Importantly, such combination therapies are associated with adverse effects like diarrhea, nausea, vomiting, epigastric pain, eosinophilia, rash, headache, dizziness, hepatotoxicity, photosensitivity, and QTc interval prolongation which can lead to treatment discontinuation [12]. 

Fluoroquinolones are broad-spectrum synthetic antimicrobial agents that exert their bactericidal effect by targeting the bacterial DNA gyrase and topoisomerase IV which inhibits bacterial DNA synthesis leading to rapid bacterial death [13]. They are active against Gram-negative and Gram-positive bacteria, anaerobes, mycobacteria, and atypical pathogens. As earlier fluoroquinolones have activity against M. tuberculosis, their use is not recommended for the treatment of CABP in India due to the risk of resistance development in this pathogen particularly in the vast majority of the population infected with latent or active TB [14]. 

In this context, levonadifloxacin, a novel benzoquinolizine subclass of quinolone antibiotic, active against both typical and atypical respiratory pathogens, does not possess any therapeutically relevant activity against *M. tuberculosis* and therefore does not pose a risk of resistance development in this pathogen [15]. Levonadifloxacin has a unique mechanism of action of targeting primacy to DNA gyrase in S. aureus leading to a potent activity against quinolone-resistant MRSA [16,17]. Several non-clinical and clinical studies have established an excellent safety profile of levonadifloxacin with lack of potential adverse effects, such as phototoxicity, QTc interval prolongation, hepatotoxicity, and nephrotoxicity. Due to its high concentrations in epithelial lining fluid and alveolar macrophages of the lungs, levonadifloxacin is a better-suited antimicrobial agent (AMA) for the treatment of respiratory infections caused by extracellular and intracellular pathogens [15,18]. Levonadifloxacin has been approved in India and is available in both IV and oral form (alalevonadifloxacin) with a comparable Pharmacokinetic profile which allows an easy step-down option and helps lower the burden on hospital infrastructure [19,20]. An excellent safety profile of levonadifloxacin coupled with comprehensive activity against all CABP pathogens makes it a better option for empirical treatment of CABP.

In this real-world, investigator-initiated, multi-center study, we compared the efficacy and safety of oral and/or IV levonadifloxacin for the treatment of adults with CABP.

## Materials and methods

Study design and participants

This prospective, open-label, single-arm, multi-centric, investigator-initiated study was conducted for the assessment of the efficacy and safety of levonadifloxacin (IV and/or oral) in CABP patients at eight healthcare centers across India. The dose regimen of levonadifloxacin was 800 mg, 90-min infusion, every 12 hours and the dose regimen of alalevonadifloxacin was 1000 mg, every 12 hours. Eligible participants were adults (aged 18 years and above) of any sex diagnosed with CABP, who provided informed consent and took no other medicines apart from the study medicines within 30 days prior to enrolment. The definition of CABP was in accordance with the standard International Classification of Diseases (ICD-10) criteria.

Ethical compliance

This study was registered with the clinical trials registry of India (CTRI/2020/11/029258) and was conducted in compliance with the protocol, the Declaration of Helsinki [21], and the International Council for Harmonisation of Technical Requirements for Pharmaceuticals for Human Use Good Clinical Practice Guidelines [22]. The study was approved by the Ethics Committee of GMERS Medical College and Hospital (RT/10/2021), Altezza Institutional Ethics Committee, Mahatma Gandhi Medical College and Research Institute (MGMCRI/2021/(07)/IHEC/21), Hansa Clinic and Hospital (HCH-EC_01/20211205), Charak Hospital and Research Centre, Narayana Diagnostics (NIEC/INDT/APP/28/11/20-02), KD Hospital, Ahmedabad (Ref. No./08/KD/IEC/2020) and Ashirwad Hospital. All participants gave their written informed consent for participation in the study.

Data collection

Data was collected and recorded in a study-specific data capture tool from the participating sites based on socio-demographic characteristics, physical examination, and systemic examination data. Patient information about their clinical condition on admission, pre-existing comorbidities, complications, and details of other treatments (including antimicrobial agents) received was also recorded.

Study outcome

The day of the first dose of levonadifloxacin (oral or IV) was considered as day one of the study. The primary endpoints were assessed as clinical outcomes on the fourth day after the first dose, at the end-of-treatment or EOT (seventh day to 14th day after the first dose), and at test-of-cure visit (TOC, 7±1 days from the EOT). Clinical responses were categorized as clinical improvement, clinical cure, clinical failure, and clinical Indeterminate. Clinical improvement was defined as ≥1 level improvement (e.g., severe to moderate) in at least two of the symptoms of CABP with no worsening by ≥1 level in other CABP symptoms. Clinical cure was defined as total resolution of all signs and symptoms of CABP (cough, purulent sputum, dyspnea, pleuritic chest pain), or improvement to such an extent that further antimicrobial therapy was not necessary. Clinical failure was defined as the persistence or worsening of signs/symptoms, the need for additional antibiotics, new pulmonary infection, progression of the chest radiograph, or death due to pneumonia. Clinical response was considered indeterminate when the evaluation was not possible due to lost to follow-up, major protocol violation, or missing data. Symptoms of CABP like cough, purulent sputum production, dyspnea, pleuritic chest pain, etc., were evaluated on a 4-point scale of absent, mild, moderate, or severe. The secondary endpoint was the microbiological response at the EOT. 

Microbiological cure was defined as the absence of a baseline pathogen with clinical improvement. Microbiological failure was the persistence of baseline pathogen and/or clinical failure. The subject was termed to be microbiologically indeterminate if the evaluation was not possible and/or clinical indeterminate. The subject must receive at least four doses of the study drug by day 3 before the investigator can consider the subject to be a clinical failure. Safety of levonadifloxacin was assessed based on findings of clinical examination, adverse events (AEs) reported by the patients, 12-lead electrocardiogram, and laboratory assessments for hemogram, serum biochemistry (random blood glucose, electrolytes, hepatic function tests, renal function tests, and urine examination) at baseline and at TOC.

Statistical analysis

The sample size was formally calculated, and data were expected to be collected over a period of eight months (January 2021 to August 2022) from eight sites. Data were summarized using descriptive statistics (number of subjects [n], mean and standard deviation [SD]) for continuous variables, and using frequency and percentage (i.e., number and proportion of subjects - n, %) for discrete/categorical variables unless specified otherwise. All statistical tests were conducted at the 5% significance level unless indicated otherwise. The data were entered in a Microsoft Office 365 Excel (Microsoft Corporation, Redmond, USA) worksheet. Statistical analysis was performed using SAS software version 9.4 (SAS Institute Inc., Cary, NC). Efficacy assessment was done using the modified intention-to-treat (mITT) analysis dataset, whereas safety assessment was done using the ITT analysis set.

## Results

There were 99 patients, found eligible (ITT set) for the study and received at least one dose of the study drug (oral alalevonadifloxacin or/and IV levonadifloxacin). However, only 74 patients completed the treatment and were included in the mITT set. Table 1 presents the baseline characteristics of these 74 patients and also provides the breakup of patients who received all oral (alalevonadifloxacin) or all-IV (levonadifloxacin) or IV followed by oral therapies. Patients with a wide range of ages (20 to 80 years) consented to be part of this study. Out of them, 23% were ≥60 years old. Patients were predominately male (68.9%).

**Table 1:** Baseline characteristics of patients (N = 74) in the modified intent-to-treat (mITT) set and treatment with levonadifloxacin and/or alalevonadifloxacin n: Number of patients; SD: Standard deviation

Parameter	n (%)	Mean (SD)	Minimum	Maximum
Age (years)	-	50.65 (15.7)	20	80
Patients with age ≥60 years	17 (22.97)	-	-	-
Body mass index (Kg/m^2^)	-	25.14 (4.6)	13.4	38.41
Gender				
Male	51 (68.9)	-	-	-
Female	22 (29.7)	-	-	-
Transgender	1 (1.4)	-	-	-
Type and duration of therapy				
All oral(alalevonadifloxacin) therapy	49 (66.2%)	7.12 (2.12) days	4 days	14 days
All IV (levonadifloxacin) therapy	8 (10.8%)	7.25 (2.71) days	4 days	13 days
IV followed by oral therapy	17 (23.0%)	10.94 (3.73) days	7 days	19 days

Consistent with real-world practice of preference for oral antibiotic therapy for CABP, 66.2% of patients in the present study received all-oral antibiotic therapy, 23% of patients received IV followed by oral switch and 10.8% of patients received all-IV treatment. In the cohort of all-oral therapy, the treatment duration was four to 14 days with a mean of 7.12 days, and an almost similar duration of treatment was observed in all-IV therapy. The treatment duration in the cohort of IV followed by oral switch was seven to 19 days with a mean of 10.94 days, suggesting a relatively prolonged treatment duration.

Regardless of the duration of therapy, clinical assessments were undertaken on the fourth day after the first dose, EOT and TOC. As depicted in Table 2, on the fourth day after the first dose, clinical improvement was achieved in 49/74 patients (66.21%) and clinical cure in 7/74 patients (9.45%). Clinical cure was documented in 69/71 (97.18%) patients at EOT in 63/63 (100%) patients at the TOC.

**Table 2:** Clinical response assessment of patients on day 4, at EOT, and at TOC mITT: Modified intent-to-treat; EOT: End of treatment; TOC: Test of cure

Clinical outcome in ‘n’ (%)	Day 4 (n=74)	Day 4 (n=71)	Day 4 (n=63)
Clinical improvement	49 (66.21%)	-	-
Clinical cure	7 (9.45%)	69 (97.18)	63 (100)
Clinical failure	1 (1.35%)	2 (2.82)	0 (0)
Indeterminate	17 (22.97%)	0 (0)	0 (0)

At the EOT, the microbiological cure was reported in 67/71 (94.36%) patients.

Patients who received only oral or only IV or IV followed by oral treatments of levonadifloxacin tolerated the drug well. There was no treatment-linked death, serious adverse effects, or adverse effect-led discontinuation. There were no treatment-linked significant changes in hematology, serum chemistry, coagulation, vital signs, or ECGs. AEs were reported in 7/92 subjects (7.6%) which included nausea, vomiting, headache, myalgia, and rash. All the AEs were mild in severity and resolved without any sequelae. In these patients, no change in study medication was required.

## Discussion

CABP is the third leading cause of death worldwide and the first leading cause of death in low-income countries [23]. *S. pneumoniae* is a common bacterial pathogen; however, atypical bacteria are also frequently encountered in CABP.

This observational study evaluated the clinical efficacy and safety of levonadifloxacin and its oral prodrug alalevonadifloxacin in the treatment of CABP. The study included 99 patients in the ITT set, with 74 patients completing the treatment and being available for the mITT analysis. The results demonstrated that levonadifloxacin, administered either orally, intravenously, or through an IV-to-oral switch, achieved a high clinical cure and was well-tolerated across various patient demographics and clinical presentations.

The baseline characteristics of the study population revealed a diverse group of patients, with ages ranging from 20 to 80 years.

Reflecting real-world clinical practice, the majority (66.2%) of patients received all-oral antibiotic therapy, aligning with the preference for oral administration in managing CABP. The duration of treatment varied, with an average of approximately seven days for both all-oral and all-IV therapies, while the IV-to-oral switch cohort had a slightly longer average treatment duration of 10.94 days. This extended duration for the IV-to-oral group may suggest a more cautious approach in transitioning from IV to oral therapy in more severe or complicated cases.

The clinical outcomes were highly favorable (>95%), with a steady increase in the clinical cure rate from the EOT to TOC.

The high efficacy observed for levonadifloxacin is mainly driven by the high lung penetration [20]. The concentration of levonadifloxacin for the entire dosing interval in the epithelial lining fluid and the alveolar macrophage remains well above the MIC90 of levonadifloxacin against both typical and atypical respiratory pathogens [17,24-26]. 

Furthermore, non-clinical studies with levonadifloxacin have shown very good immunomodulatory or anti-inflammatory activity. Levonadifloxacin significantly reduced the inflammatory responses in human whole-blood assay through inhibition of proinflammatory cytokines and in the acute lung injury model by lowering lung total white blood cell count, myeloperoxidase, and cytokine levels (TNF-alpha, IL-6, and IL-1). This finding was corroborated using histopathological evidence that suggested minimal infiltration of neutrophils into the lung tissue by levonadifloxacin after a lipopolysaccharide challenge [27].

Levonadifloxacin is not a substrate for NorA efflux pump; hence, the development of antimicrobial resistance by efflux mechanisms by bacteria is minimized [24]. Also, it has activity against slow-growing or intra-cellular staphylococci and has improved activity in acidic pH [15].

The excellent oral bioavailability of levonadifloxacin not affected by food, coupled with its favorable pharmacokinetic and pharmacodynamic profile, makes it a preferred antibiotic for varied infections [16].

Levonadifloxacin and alalevonadifloxacin were well-tolerated across all patient groups. The absence of treatment-linked deaths, serious adverse effects, or discontinuations due to adverse effects underscores the favorable safety profile of these antibiotics. The observed AEs (vomiting, nausea, myalgia, rash, and headache) were mild and resolved without sequelae, not necessitating any changes in study medication. An excellent safety profile with a lack of potential adverse effects, such as phototoxicity, prolongation of QTc interval [28], hepatotoxicity, and nephrotoxicity, could be a better option for the treatment of CABP.

The findings from this study suggest that levonadifloxacin, available in both oral and IV formulations, provides a versatile and effective treatment option for CABP. The ability to switch from IV to oral therapy without compromising efficacy offers significant advantages in terms of patient convenience and healthcare resource utilization. The drug's favorable safety profile, even among older adults and those with underlying comorbidities, supports its use as a reliable treatment option in diverse clinical scenarios.

The study was conducted across multiple centers, enhancing the generalizability of the findings and reducing the impact of center-specific biases. This study comprehensively evaluated clinical outcomes, offering a holistic understanding of the treatment efficacy of levonadifloxacin. The reported low incidence of AEs associated with levonadifloxacin indicates its favorable safety.

The lack of a comparative arm limits the ability to draw direct comparisons with other treatment modalities.

## Conclusions

Levonadifloxacin, whether administered orally, intravenously, or through an IV-to-oral switch, demonstrates high clinical efficacy and a favorable safety profile in the treatment of adults with CABP. These findings highlight its potential as a versatile and effective antibiotic therapy for managing CABP in a diverse patient population. Features of levonadifloxacin, such as availability in both IV and oral forms, minimal drug-drug interactions, and lack of the need to adjust dosages in renal and hepatically impaired patients along with a broad spectrum of coverage, make it a suitable agent that meets several unmet clinical needs of physicians. Levonadifloxacin would enable the clinicians to easily switch over from injectable to oral formulation allowing early discharge from the hospital.
